# Application of Functional Biocompatible Nanomaterials to Improve Curcumin Bioavailability

**DOI:** 10.3389/fchem.2020.589957

**Published:** 2020-10-06

**Authors:** Ziyun Li, Mingfei Shi, Ning Li, Ruodan Xu

**Affiliations:** ^1^Institute of Basic Theory for Chinese Medicine, China Academy of Chinese Medical Sciences, Beijing, China; ^2^The Third School of Clinical Medicine, Nanjing University of Chinese Medicine, Nanjing, China

**Keywords:** curcumin, bioavailability, nano-formulation, biocompatible, therapeutic application

## Abstract

Curcumin is a lipophilic natural product extracted from turmeric and commonly used as a dietary spice. Being multi-functional, curcumin has been proposed in the prevention and treatment of a broad spectrum of diseases. However, due to unsatisfactory aqueous solubility and hence low bioavailability, clinical application of curcumin has been greatly restrained. To break these limitations, biocompatible nanoformulation, such as liposomes, nanoparticles, micelles, nanoemulsions and conjugates has been employed as alternatives to improve *in vivo* delivery of curcumin. In this scenario, in order to enhance bioavailability of curcumin, the choice of effective molecules as facilitators is of prominence. In this review, we focus on functional biocompatible materials, including polymers, protein molecules, polysaccharide, surface stabilizers and phospholipid complexes, and decipher their potential applications as how they assist to promote medicinal performance of curcumin.

## Introduction

Curcumin (1,7-bis(4-hydroxy-3-methoxyphenyl)-1,6-heptadiene-3,5-dione), is a natural polyphenol product found in the rhizome of turmeric plant (*Curcuma longa L*.) native from southern Asia (Willenbacher et al., 2019). In turmeric, curcumin is identified as the major active curcuminoids, as compared to its two dimethoxy derivatives, dimethoxycurcumin (DMC, one -OMe at the outer phenol rings is removed) and bis-demethoxycurcumin (BMC, two -OMes are removed), with the ratio being 77:3:17 (Ma et al., 2019). Over the past few decades, a plenty of curcumin bioactivities have been revealed, including anti-cancer (Nagaraju et al., 2019; Yedjou et al., 2019), anti-inflammation (Farhood et al., 2019), antioxidation (Abrahams et al., 2019), anti-microbe (Sharifi et al., 2020), anti-hepatotoxicity (Fujiwara et al., 2008) and anti-Alzheimer (Bhat et al., 2019), and this wide range of biological effects has been attributed to essential molecules related to human diseases, such as cyclooxygenase-2 (COX-2), matrix metallopeptidases (MMPs), glutathione, protein kinase C, ATPase, NF-κB, activator protein 1 (AP-1), P-glycoprotein (P-gp), multidrug resistance-associated protein 1 (MRP-1), multidrug resistance-associated protein 2 (MRP-2), and cyclin D1 (Lev-Ari et al., 2006; Stridh et al., 2010; Kao et al., 2011; Sreenivasan et al., 2012; Mishra et al., 2015; Ge et al., 2016; Lopes-Rodrigues et al., 2016; Seo et al., 2016; Zhu et al., 2016). However, when evaluating curcumin in human subjects, therapeutic outcomes of curcumin remain largely unsatisfactory even at a single high dose of up to 12 g (Kesisoglou et al., 2007), although no adverse events have been reported. The reason behind limited benefits *in vivo* is assumedly associated with its low bioavailability due to poor absorption and rapid systemic elimination when applied as its raw insoluble form. Indeed, curcumin is poorly soluble in water (11 ng/ml, pH 5.0), and it has been shown that only the soluble curcumin can be absorbed by intestinal epithelial cells in the gastrointestinal tract (GI) (Ireson et al., 2002). Although organic solvents, oils and surfactants have been suggested to produce highly soluble solutions of curcumin, their cytotoxic effects exert additional considerations in the development of curcumin-originated medications (Tønnesen et al., 2002). Moreover, the fact that curcumin is highly sensitive to light and metabolism of curcumin is under the control of several GI factors, such as pH, gastric acid, bile, and digestive enzymes (Ma et al., 2019), further hampers its therapeutic application (Rutz et al., 2019).

**Figure 1:**
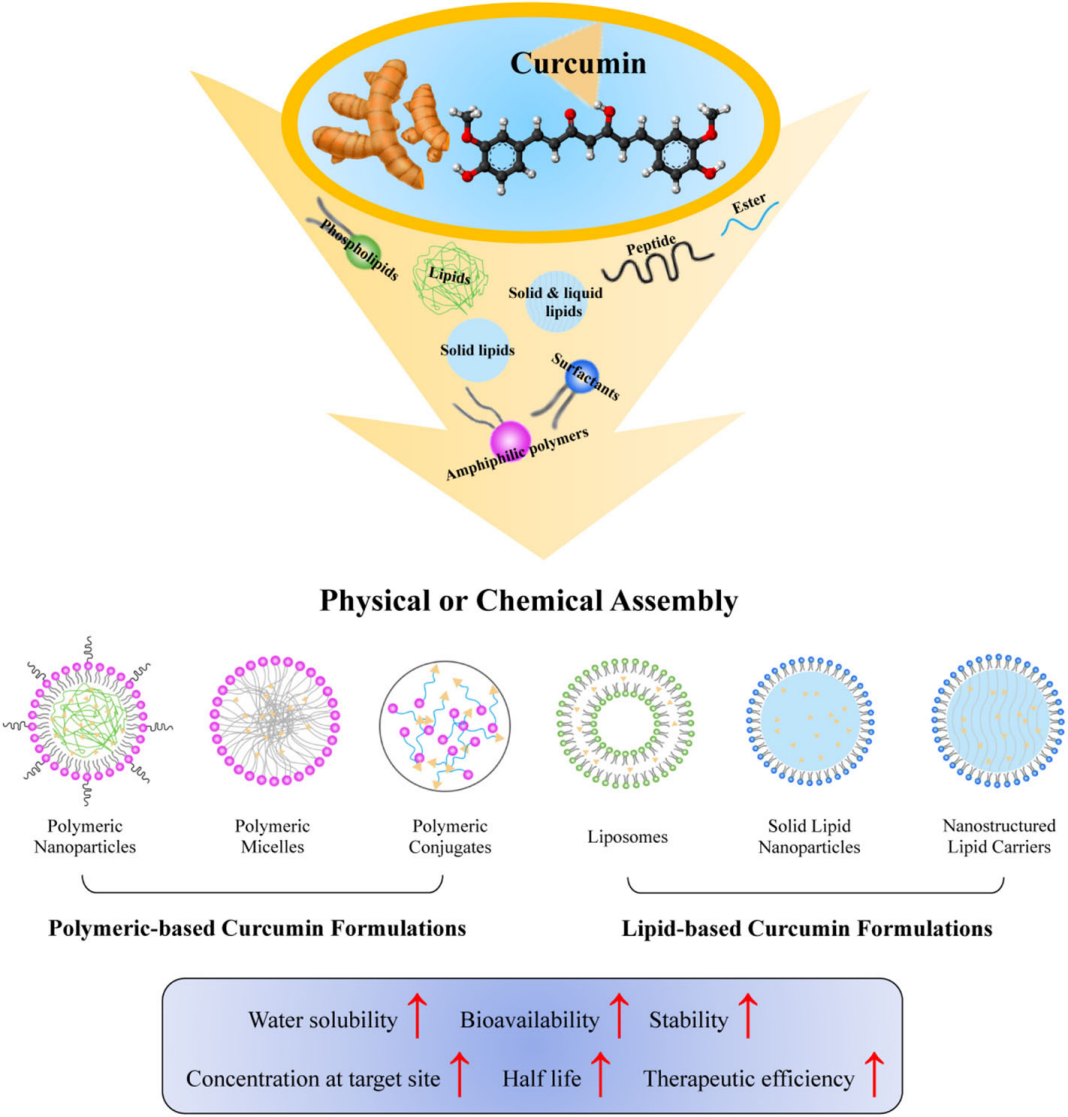
Functional biocompatible nanomaterials employed to improve curcumin bioavailability. Biocompatible materials, including phospholipids, amphiphilic polymers, surfactants, peptides, solid lipids, liquid lipids, and ester have been incorporated with curcumin by physical or chemical assembly to form various nanoformulations of curcumin. The resultant nanocurcumin has improved water solubility, bioavailability, stability, concentration at target site, half-life, and therapeutic efficiency of curcumin.

With the ongoing development of nanotechnology, several strategies, and approaches have been employed to improve the solubility and bioavailability of curcumin (Sun et al., 2012). Based on specific aims or requirements for curcumin, utilization of organic nanomaterials including polymers, lipids, dendrimers, and polysaccharide with functionalization of targeted therapy has been proposed and studied. By incorporating with nano-technologies, the resultant curcumin has been efficiently scaled down to nanometer, which greatly helps to maximize the bioactivities and to minimize the physical and chemical degradation of curcumin (Mohanty et al., 2012). The initiation of using nanomaterials and their successful management in physicochemical properties of curcumin *in vivo* dramatically triggered a rapid progress of curcumin in industrial and therapeutic applications. This review focuses on capacities of different biocompatible nanomaterials and approaches for various types of curcumin formulations, as summarized in both Figure 1, Tables 1, 2, in order to better understand, use, develop, and eventually assist to formulate novel curcumin products for medical purposes.

**Table 1:** Characteristics of different nanocurcumin formulations.

**Types of nanocurcumin formulations**	**Publication year**	**Materials**	**Preparation methods**	**Size (nm)**	**PDI**	**Zeta potential (mV)**	**Encapsulation efficiency (%)**	**Stability**	**Biological model**	**Biodistribution**	**Bioavailability**	**Reference**
Polymeric curcumin nanoparticles	2016	Eudragi E100 Poloxamer 188	Emulsification diffusion evaporation method	248.40 ± 3.89	0.212 ± 0.013	+ 28.9 ± 0.47	65.77 ± 3.17	Remained stable for 3 months at room temperature.	Colon-26 tumor-bearing BALB/c mice and Albino Wister rats	NA	AUC: 95-fold increased Cmax: 90.82-fold increased t_1/2_: 3.84-fold extended	(Chaurasia et al., 2016)
	2018	Saponin	PH-driven method	51.9 ± 3.0	0.242 ± 0.045	−30.44 ± 0.43	91.8 ± 2.8	Remained stable at 4° or 25°C for 1 month (pH 6.5).	SD Rats	NA	AUC: 8.9-fold increased Cmax: 14.7-fold increased	(Peng et al., 2018b)
	2019	Tri-CL-mPEG Poloxamer 188	Microchannel technology	116 ± 3	0.197 ± 0.008	−12.2 ± 0.404	91.42 ± 0.39	Remained stable at 4°C for 60 days.	Kunming mice.	Heart: 6.05%Liver: 13.93%Spleen: 26.39%Lung: 37.52%Kidney: 16.11%	AUC: 4.14-fold increased Cmax: 12.4-fold increased t_1/2_: 2.07-fold extended	(Wu et al., 2019)
Polymeric curcumin micelles	2011	PLGA, PEG	Ring-opening method	26.29	NA	−0.71	70 ± 0.34	NA	Kunming mice	Liver: 6.41%Spleen: 2.5%Lung: 80.98%Brain: 9.98%	AUC:1.31-fold increased t_1/2_: 2.48-fold increased	(Song et al., 2011)
	2017	mPEG, PCL	NAnoprecipitation method	81.0	0.172	−11.5	89.32 ± 0.34	NA	Male adult Wister rats	NA	AUC: 7.51-fold increased Cmax: 52.8-fold increased t_1/2_: 4.63-fold increased	(Manjili et al., 2017)
	2018	Sophorolipid	PH-driven method	60.8 ± 3.7	0.295 ± 0.045	−41.2 ± 2.0	82.2 ± 0.7	Remained stable at pH values (3–8) for 30 min and aggregated at PH values (1.5–2).	SD Rats	NA	AUC: 4.55-fold increased Cmax: 5.83-fold increased	(Peng et al., 2018a)
	2019	Vitamin E mPEG2k-DSPE	NAnoprecipitation method	29.84 ± 0.89	0.261 ± 0.06	−21.5 ± 1.8	96.7	Improved stability in phosphate buffer saline (PBS) (pH = 7.4), 10% FBS culture medium and rat plasma.	SD Rats	NA	AUC: 107-fold increased t_1/2_: 10.6-fold increased	(Zhang et al., 2019)
Polymeric curcumin conjugate	2014	Cholesteryl chloroformate hyaluronic acid	Esterification reaction	29.2 ± 5.4	0.4 ± 0.1	−38.4 ± 3.9	NA	Remained stable in SGF and SIF, only 5% released in SGF after 4 h, less than 20 and 10% released in SGF and SIF after 48 h	Human pancreatic adenocarcinoma MiaPaCa-2 xenograft model bearing female nu-nu mice	Curcumin distributed in livers and pancreatic tumors was significantly higher than that in kidneys	13-fold tumor suppression t_1/2_: over 20-fold increased	(Wei et al., 2014)
Curcumin liposome complex	2017	DMPC DMPG Cholesterol DSPE-PEG2000 HA	Thin film evaporation	236.4 ± 5.2	0.232	−36.8 ± 1.9	65.8 ± 3.3	Remained stable over 4 weeks at 4°C.	BALB/c mice	NA	AUC: 926-fold increased	(Sun et al., 2017)
	2018	L-α-phosphatidylcholine Chitosan	Thin-film dispersion method and electrostatic adsorption method	161.6 ± 12.3	0.18 ± 0.04	−30.2 ± 0.13	NA	Remained stable after 14 days at 4°C.	SD Rats	CMCS/TMC-LPs distributed more curcumin than TMC-LPs in spleen, liver, and lung	AUC: 13.05-fold increased Cmax: 3.48-fold increased	(Tian et al., 2018)
Solid lipid nanocurcumin	2017	N-carboxymethyl chitosan Glyceryl monostearate Soya lecithin Poloxamer 188	Hot homogenization and sonication method	245.1 ± 5.4	0.295 ± 0.16	−10.4 ± 3.9	78.5 ± 3.1	Remained stable at room temperature for 60 days.	SD Rats	NCC-SLN exhibited more curcumin than C-SLN in intestine	AUC: 8.89-fold increased Cmax: 3.24-fold increased	(Baek and Cho, 2017)
	2020	Tristearin PEG100SE	Ethanolic precipitation and ultraturrax homogenization	147.80 ± 1.90 (PEG100SE concentration: 46.9 mM)	NA	−1.18 ± 0.39	92.55 ± 0.20	Remained stable under high ionic strength or acidic conditions.	SD Rats	NA	AUC: 6-fold increased Cmax: 6-fold increased	(Ban et al., 2020)
NAnostructured lipid curcumin	2017	Cholesterol oleate Glycerol trioleate S100-TCA EDCI Phosphatidylcholine	Solvent evaporation method	99.6 ± 5.9	0.26 ± 0.107	−3.54 ± 0.50	96.59 ± 0.08	NA	SD Rats	NA	AUC: 24-fold increased Cmax: 49.54-fold increased	(Tian et al., 2017a)
	2017	PEG(100)-monostearate N-acetyl-L-cysteine Cholesterol oleate Glycerol trioleate Phosphatidylcholine	Solvent evaporation method	89.2 ± 1.5	0.24 ± 0.098	−9.55 ± 0.39	95.68 ± 0.04	NA	SD Rats	C6-NAPG100-NLC distributed more curcumin in the duodenum, jejunum, and, ileum compared with NLC	ACU: 499.45-fold increased Cmax: 941.36-fold increased	(Tian et al., 2017b)

*PDI, polydispersity index; AUC, area under the curve; Cmax, maximum concentration; t_1/2_, half-life; Tri-CL-mPEG, tricarballylic acid-poly (ε-caprolactone)-methoxypolyethylene glycol; mPEG2k-DSPE, N-(Carbonyl-methoxypolyethyleneglycol 2000)-1,2-distearoyl-sn-glycero-3-phosphoethanolamine; PEG, polyethylene glycol; PCL, poly (ε-caprolactone); SD Rats, Sprague–Dawley Rats; CMCS/TMCLPs, Carboxymethyl chitosan (CMCS) and quaternary ammonium chitosan (TMC)-coated liposomes; NAPG, N-acetyl-L-cysteine-polyethylene glycol (100)-monostearate; NLC, nanostructured lipid carrier; NCC-SLN, N-carboxymethyl chitosan (NCC) coated curcumin-loaded SLN; SGF, simulated gastric fluid; SIF, simulated intestinal fluid; DMPC, 1,2-Dimyristoyl-sn-glycero-3-phosphocholine; DMPG,1,2-dimyristoyl-sn-glycero-3-phospho-rac-(1-glycerol); DSPE, 1,2-distearoyl-sn-glycero-3-phospho-rac-glycerol; HA, hyaluronic acid; S100-TCA, taurocholic acid–polyethylene glycol 100–monostearate; NA, non-available*.

**Table 2:** Comparison of recent approaches used in nanocurcumin formulations.

**Types of nanocurcumin formulations**	**Methods**	**Advantages**	**Disadvantages**
Polymeric-based curcumin formulations	Nanoprecipitation	Easy preparation Scaling up for industrial production	Selection of appropriate solvent and polymers
	Emulsification-solvent diffusion	High curcumin loading Narrow size distribution and uniform size Scaling up for industrial production	High volume of water
	High-pressure homogenization	Batch-to-batch control	Pressure and homogenization- dependent particle size High-pressure homogenizer request
	pH-driven method	Simple and fast No organic solvents	Water solubility and chemical stability of the curcumin dependent
	Esterification reaction	High curcumin loading Controlled curcumin's solubility, stability, and release	Insufficient reaction By-products
Lipid-based curcumin formulations	Thin film hydration technique	Easy operation	Large size and size distribution Non-uniform lipid layers
	Reverse phase evaporation	High curcumin loading	Large size
	Solvent evaporation	Low toxicity	Complex influencing factors: stirring speed, homogenizer type, and polymer concentration
	Microfluidization method	Batch-to-batch control High homogeneity and small size	Individualized parameters

## Types of Nanocurcumin Formulations

### Polymeric-Based Curcumin Formulations

#### Polymeric Curcumin Nanoparticles

Polymeric nanoparticles (NPs) have been widely studied in the field of drug delivery due to their high capacity of drug loading, controlled release, and efficient degradation. Curcumin-loaded polymeric NPs are generally colloidal particles in spherical or core-shell structures with a size range of 60–1,000 nm. Both natural and synthetic polymers have been intensively investigated for NPs formulation, in which natural polymers, such as polysaccharides [alginate, starch, chitosan, hyaluronic acid (HA), etc.] and proteins (collagen, albumin, fibrin, silk, etc.), showed high biocompatibility and biodegradability *in vivo*. Nevertheless, their significant batch-to-batch variation makes the extensive following purification procedure unavoidable to reach bio-similarities (Karlsson et al., 2018). In this respect, biodegradable synthetic polymers such as polyethylene glycol (PEG), polylactide (PLA), polyglycolide acid (PGA), poly (ε-caprolactone) (PCL), and polylactic-co-glycolic acid (PLGA), with the advantage of simple control in large-scale production are gaining an increasing importance. Although holding a promise, other interfering factors, such as potential cytotoxicity or immunogenicity have been shown introduced by products or metabolites from their unexpected degradation (Sun et al., 2012). Typically, zein is a water-insoluble amphiphilic protein from the corn, which is commonly used in curcumin encapsulation (Hong et al., 2018). Chen et al. developed curcumin-zein-HA NPs by incorporation of curcumin in a hydrophobic zein core with a hydrophilic HA shell using layer-by-layer electrostatic deposition. This formulation prolonged the release of curcumin with the increased HA surface density, and concomitantly offered a 3-fold increase in light and thermal stability compared to free curcumin. However, the HA hydrophilic coating surface may result in insufficient NPs cellular uptake and transport (Chen et al., 2019). To solve this problem, Akhtar et al. prepared curcumin-chitosan NPs by ionic gelation, in which curcumin cellular uptake was triggered by positive chitosan electrostatic interaction with negative intestinal mucin glycoproteins. Curcumin-chitosan NPs by oral administration have led to 6.4-fold increase in blood half-life and excellent penetration ability across transmucosal barrier (Akhtar et al., 2012). Another example falls on PEG, which is the most widely used hydrophilic polymer to prepare curcumin-loaded NPs for tumor therapy. Generally, PEG conjugates take the advantage of enhanced permeability and retention (EPR) effect and get accumulated in tumor vessels through leaky vasculature and poor lymphatic drainage. To increase tumor targeting specificity and avoid high-dose curcumin-induced toxicity in non-tumor tissues, Guo et al. fabricated a dual functional MMPs-responsive curcumin-loaded nanoparticles (Cur-P-NPs) based on a tri-block biomaterial (mPEG-Peptide-PCL) through solvent evaporation. Besides PEGylation, peptide (ACP)-GPLGIAGQr9-(ACP) was also designed to target matrix metalloproteinases-2 (MMP-2), which is often up-regulated in tumor cells. In this study, Cur-P-NPs with a diameter of 159.7 nm and encapsulation efficiency of 80.12% selectively targeted and penetrated A549 non-small cell lung cancer cells, when compared with that in non-targeted L929 mouse fibroblast cells (Guo et al., 2018). Further efforts to improve the accuracy of curcumin delivering have been made by designing polymers whose target or release is environmentally triggered (such as pH, temperature, light, enzymes, and biomolecules). Poly (2, 4, 6-trimethoxybenzylidene-1,1,1-tris(hydroxymethyl)ethane methacrylate) (PTTMA) are pH-sensitive polymers undergoing hydrophobic-to-hydrophilic transition in mildly acidic environment due to the hydrolysis reaction of acid-labile cyclic benzylidene acetal groups (CBAs). Disulfide bridge (SS) between polymer chains are used as a reduction-responsive linkage for structure disassembles. Zhao et al. prepared curcumin-loaded PTTMA-g-SS-PEG NPs by reversible addition-fragmentation chain transfer polymerization (RAFT) copolymerization and coupling reaction. A particle of 130.2 nm in size and encapsulation efficiency of 96% was obtained. The curcumin-loaded PTTMA-g-SS-PEG NPs were found more stable (< 15% release) in normal physiological conditions, whereas upto 76.8 and 94.3% release of curcumin were achieved in acidic and acidic with reductive condition, respectively. Functionally, curcumin-loaded PTTMA-g-SS-PEG NPs effectively inhibited the proliferation of both esophageal carcinoma cells and human liver cancer cells (HepG-2). Therefore, these data point out that dual pH and reductive-responsive materials could facilitate a rapid release of curcumin in tumor cells, which is more acidic than normal cells (Zhao et al., 2013).

#### Polymeric Curcumin Micelles

Polymer micelles are generally formed by amphiphilic block copolymers due to hydrophobic interaction in a core-shell structure with a very narrow size range from 10 to 100 nm in aqueous media (Torchilin, 2007). Based on these properties, polymer micelles served as transporters for curcumin to evade the mononuclear phagocyte system (MPS) and increase curcumin accumulation at diseased tissues (Moghimi et al., 2005; Oerlemans et al., 2010; Hanafy et al., 2018; Hussein and Youssry, 2018). Aiming to achieve long blood-circulating micelles, Kumar et al. fabricated a mixed micellar system encapsulation of curcumin, in which short chain of PEG15-hydroxystearate together with long PEG chain of D-α-tocopheryl polyethylene glycol succinate1000 (TPGS1000) were used to create a stronger interfacial bond (Kumar et al., 2017). Although promising, the actual shear flow led to alterations of polymer structure or conformation in blood, which drove micelles dissociation and thus drug release before reaching the targeted organs (Almeida et al., 2013). In order to obtain more stable curcumin micelles, Li et al. formulated amphiphilic Pluronic F-127 (PF127) micelles to encapsulate curcumin by thin-film evaporation method. PF127-curcumin-micelles are spherical, with a diameter of 25.4 ± 1.8 nm and 91.76 ± 5.93% encapsulate efficiency. *In vitro* drug release showed a biphasic release profile with an initial release of 18% at 24 h and 50% sustained drug release over 9 days. *In vivo* study in mice further confirmed that the bioavailability of PF127-curcumin-micelles was enhanced up to 1.69-fold than that of free curcumin (Li et al., 2014).

#### Polymeric Curcumin Conjugates

In addition to physical encapsulation, chemical modifications of curcumin by polymer conjugates have also been investigated, which are advantageous in efficient curcumin loading and controlled release (Alves et al., 2018). The appropriate choice of polymer and the drug conjugate linkage can be manipulated to control curcumin's solubility and stability. Chemically, the two phenolic rings and active methylene groups of curcumin provide potential sites to conjugate with water soluble polymers, including HA, alginate and hydroxyethyl starch (HES) *via* esterification reactions (Yallapu et al., 2012). Dey and Sreenivasan developed alginate-curcumin conjugate by covalently conjugating curcumin to the C-6 carboxylate functional group of hydrophilic sodium alginate *via* an ester linkage to enhance solubility and stability at physiological pH of curcumin (Dey and Sreenivasan, 2014). Likewise, the ester linkage can be cleaved quickly by acid or esterase, thus the release pattern of curcumin can be achieved, and therefore fulfill the effectiveness of the curcumin (Fleige et al., 2012). For instance, recent work by Chen et al. fabricated a HES-curcumin conjugates through conjugating HES to curcumin *via* an acid-labile ester linker. This formulation increased solubility of curcumin to a thousand times higher than free curcumin, meanwhile offered curcumin with protections against UV and thermal degradation. In addition, adjusting aqueous media to a lower pH led to increased curcumin release and improved efficacy correspondingly, as evidenced by its anticancer and antioxidant activity in HeLa cervical carcinoma cells and Caco-2 human colorectal adenocarcinoma cells (Chen et al., 2020).

### Lipid-Based Nanocurcumin

#### Curcumin Liposome Complex

Liposomes are nanosized spherical vesicles comprising phospholipid bilayers, and are created by physiologically accepted natural or synthetic phospholipids (Papahadjopoulos and Kimelberg, 1974). In aqueous media, phospholipid could self-assemble into a bilayer structure due to itself amphiphilic property with the basis of a hydrophilic head group and two hydrophobic acyl chains. This architecture endowed liposomes to be able to load with both lipophilic and hydrophilic drugs within phospholipid bilayers and aqueous core, respectively (Frolov et al., 2011). The selection of liposome composition and preparation method have found highly relevant to curcumin's bioavailability and efficacy. In this regard, a variety of lipids have been tested in liposomes preparations of curcumin. Thangapazham et al. prepared curcumin-loaded liposomes with lipid composition of 1,2-Dimyristoyl-sn-glycero-3-phosphocholine (DMPC), dipalmitoyl phosphatidylcholine (DPPC), or egg phosphatidylcholine (egg PC) (Thangapazham et al., 2008). Authors showed that DMPC-based liposomes allowed the greatest amount of curcumin entrapment, indicating curcumin preferentially partitioned into liposomes prepared from DMPC other than DPPC or egg PC. Besides lipids, cholesterol as a membrane constituent is widely incorporated in liposome composition to improve the liquidity of lipid membrane. Xu et al. prepared curcumin-loaded liposomes from soybean phosphatidylcholine (SoyPC) and cholesterol using the conventional thin-film dispersion method, and showed potential therapeutic effect in inhibiting cancer cells proliferation *in vitro* and *in vivo* (Xu et al., 2018). However, the clinical use of cholesterol as part of a drug is controversial due to the potential adverse effect of its oxidized products to human. In another study, Hamano et al. prepared curcumin liposomal formulation with DMPC and 5 mol% Tween 80 using microfluidic and thin-film hydration, respectively. Though, no significant difference was observed in the particle size (117.1 ± 4.57 nm) and polydispersity index (PDI, 0.120 ± 0.005) between these two liposomes preparation methods, vesicles obtained from thin-film hydration tended to form aggregates after 24 h storage at 4°C, whereas curcumin-liposome from microfluidics remained highly stable for at least 3 weeks. Moreover, in comparison to standard curcumin suspension, this curcumin-liposome improved its aqueous solubility by 700-fold, which corresponds to 8–20-fold bioavailability increased in tumor-bearing mice (Hamano et al., 2019).

#### Solid Lipid Nanocurcumin

Solid lipid nanoparticles (SLNs) are a new generation nanoemulsions developed to avoid residual organic solvents. SLNs are able to encapsulate both hydrophobic and hydrophilic drugs. Production of SLNs can be realized by solid lipids and surfactants using high-pressure homogenization (HPH), and can be modified to yield particles ranging from 10–1,000 nm in size for a large-scale production (Jenning et al., 2002). Considering the thermal effect that is likely to impact the curcumin's chemical stability, lipids with low melting point and being solid state at room or body temperature, such as monostearin, glyceryl monostearate, precirol ATO 5 (mono, di, triglycerides of C16-C18 fatty acids), compritol ATO 888, stearic acid, and glyceryl trioleate would be optimal to use (Manjunath et al., 2005). Considering surfactants involved in SLNs, by reducing interfacial tension between hydrophobic surface of lipid and aqueous environment, surfactants act as surface stabilizers. In studies to date, the most preferred surfactants in clinical applications are poloxamer 188, Tween 80 and dimethyl dioctadecyl ammonium bromide (DDAB) (Manjunath et al., 2005; Tapeinos et al., 2017), which facilitate curcumin SLNs with increased bioavailability and improved pharmacological activity. In addition to production time and speed, a proper selection of lipids (amount) and surfactants (concentration) would have significant impacts on their physiological performance. Ban et al. prepared SLNs to encapsulate curcumin using tristearin and PEGylated surfactant. Curcumin loaded in long-PEGylated [polyoxyethylene (100) stearyl ether, PEG100SE] SLNs showed greater absorption and long-term stability in rat after oral administration. In this study, bioavailability of curcumin can be increased 12-fold in PEG100SE (17.1 mM) formulated SLNs and 13.2-fold in PEG100SE (46.9 mM) formulated SLNs, whereas the short-PEGylated formulated SLNs does not (Ban et al., 2020). In another study, to overcome the initial burst release of SLNs, Huang et al. applied sodium caseinate (NaCas) and sodium caseinate-lactose (NaCas-Lac) conjugates as bioemulsifiers to stabilize curcumin SLNs. The presence of the glycated lactose on surface provided steric hindrance, which therefore enabled the dispersibility and enhanced stability of curcumin at acidic pH. By doing this, curcumin loaded NaCas-Lac SLNs enhanced antioxidant activity by 3–4-folds in relative to that of free curcumin (Huang et al., 2020).

#### Nanostructured Lipid Curcumin

Nanostructured lipid carriers (NLCs), also known as hybrid lipid nanoparticles (HLNs), are composed of solid and liquid lipids mixture (Selvamuthukumar and Velmurugan, 2012). By addition of liquid lipid, the melting point will be decreased, and an amorphous solid state will appear at ambient temperature. This unique asymmetric lipid matrix structure provides a greater load of drug as well as minimized drug expulsion (Weber et al., 2014). Sadegh et al. prepared NLCs loading curcumin with solid lipid (cetyl palmitate and cholesterol) and liquid lipid (oleic acid) using homogenization and ultrasonication, with resultant nanoparticles being 117.36 ± 1.36 nm in size and 94 ± 0.74% in encapsulation efficiency. The particle size was not physically changed after 3 months of storage, demonstrating a great storage stability of this formulation. In addition, antioxidant activity of curcumin-NLCs as evaluated by DPPH (2, 2-diphenyl-1-picrylhydrazyl) free radical scavenging efficiency was unaltered compared with free curcumin, indicating the biological property of curcumin was not affected by the production process. Moreover, *in vivo* pharmacokinetic studies in brain showed that the area under the curve (AUC) for curcumin-NLCs (AUC = 505.76 ng/g·h) was significantly higher than both free curcumin (AUC = 0.00 ng/g·h) and curcumin-SLNs (AUC = 116.31 ng/g·h) in rats exposed to 4 mg/kg curcumin intravenously (Sadegh Malvajerd et al., 2018).

## Conclusion

Great progress has been made in the development of functional biocompatible nanomaterials contributing to the versatility of nano-curcumin formulations. In particular, biodegradable materials used in nanocurcumin are advantageous in biocompatibility and biosafety, showing greater potential to achieve rapid clinical application. In each formulation, materials are chosen, and used for specific purpose in order to improve curcumin bioavailability, such as enhanced water solubility, increased loading or encapsulation efficiency, safe degradation, controlled release, and targeted therapy. Among these, it is noted that a dual or multi-purpose can be achieved with the use of same material in different formulations. In particular, biodegradable materials used in nanocurcumin have the advantages of biocompatibility and biosafety, considered to held great promise in clinical application. Meanwhile, diverse approaches to prepare nanocurcumin have been established to construct nanoparticles of different physiochemical features. Therefore, the selection of an optimal method in practice requires considerations of both strengths and weaknesses of each technique, and physiochemical properties of the desired products. In spite of complexity in formulations, low yield, and high cost are of prime importance that influence clinical translation of nanocurcumin. Thus, further investigations will be needed in order to better understand the interaction of materials with curcumin prior to developing nanomedicine in clinics.

## Author Contributions

ZL and MS contributed equally to the literature search, data integration, and writing. RX and NL instructed, wrote, and review the manuscript. All authors contributed to the article and approved the submitted version.

## Conflict of Interest

The authors declare that the research was conducted in the absence of any commercial or financial relationships that could be construed as a potential conflict of interest.

## References

[B1] Abrahams S. Haylett W. L. Johnson G. Carr J. A. Bardien S. (2019). Antioxidant effects of curcumin in models of neurodegeneration, aging, oxidative and nitrosative stress: a review. Neuroscience 406, 1–21. 10.1016/j.neuroscience.2019.02.020 30825584

[B2] Akhtar F. Rizvi M. M. A. Kar S. K. (2012). Oral delivery of curcumin bound to chitosan nanoparticles cured Plasmodium yoelii infected mice. Biotechnol. Adv. 30, 310–320. 10.1016/j.biotechadv.2011.05.009 21619927

[B3] Almeida H. Amaral M. H. Lobão P. Sousa Lobo J. M. (2013). Applications of poloxamers in ophthalmic pharmaceutical formulations: an overview. Expert Opin. Drug Deliv. 10, 1223–1237. 10.1517/17425247.2013.796360 23688342

[B4] Alves T. F. das Neves Lopes F. C. Rebelo M. A. Souza J. F. da Silva Pontes K. Santos C.. (2018). Crystalline ethylene oxide and propylene oxide triblock copolymer solid dispersion enhance solubility, stability and promoting time-controllable release of curcumin. Recent Patents Drug Deliv. Formul. 12, 65–74. 10.2174/1872211312666180118104920 29345599

[B5] Baek J. S. Cho C. W. (2017). Surface modification of solid lipid nanoparticles for oral delivery of curcumin: improvement of bioavailability through enhanced cellular uptake, and lymphatic uptake. Eur. J. Pharm. Biopharm. 117, 132–140. 10.1016/j.ejpb.2017.04.013 28412471

[B6] Ban C. Jo M. Park Y. H. Kim J. H. Han J. Y. Lee K. W.. (2020). Enhancing the oral bioavailability of curcumin using solid lipid nanoparticles. Food Chem. 302:125328. 10.1016/j.foodchem.2019.125328 31404868

[B7] Bhat A. Mahalakshmi A. M. Ray B. Tuladhar S. Hediyal T. A. Manthiannem E.. (2019). Benefits of curcumin in brain disorders. BioFactors 45, 666–689. 10.1002/biof.1533 31185140

[B8] Chaurasia S. Chaubey P. Patel R. R. Kumar N. Mishra B. (2016). Curcumin-polymeric nanoparticles against colon-26 tumor-bearing mice: cytotoxicity, pharmacokinetic and anticancer efficacy studies. Drug Dev. Ind. Pharm. 42, 694–700. 10.3109/03639045.2015.1064941 26165247

[B9] Chen S. Han Y. Huang J. Dai L. Du J. McClements D. J.. (2019). Fabrication and characterization of layer-by-layer composite nanoparticles based on zein and hyaluronic acid for codelivery of curcumin and quercetagetin. ACS Appl. Mater Interf. 11, 16922–16933. 10.1021/acsami.9b02529 30985111

[B10] Chen S. Wu J. Tang Q. Xu C. Huang Y. Huang D.. (2020). Nano-micelles based on hydroxyethyl starch-curcumin conjugates for improved stability, antioxidant and anticancer activity of curcumin. Carbohydr. Polym. 228:115398. 10.1016/j.carbpol.2019.115398 31635734

[B11] Dey S. Sreenivasan K. (2014). Conjugation of curcumin onto alginate enhances aqueous solubility and stability of curcumin. Carbohydr. Polym. 99, 499–507. 10.1016/j.carbpol.2013.08.067 24274536

[B12] Farhood B. Mortezaee K. Goradel N. H. Khanlarkhani N. Salehi E. Nashtaei M. S. (2019). Curcumin as an anti-inflammatory agent: Implications to radiotherapy and chemotherapy. J. Cell Physiol. 234, 5728–5740. 10.1002/jcp.27442 30317564

[B13] Fleige E. Quadir M. A. Haag R. (2012). Stimuli-responsive polymeric nanocarriers for the controlled transport of active compounds: concepts and applications. Adv. Drug Deliv. Rev. 64, 866–884. 10.1016/j.addr.2012.01.020 22349241

[B14] Frolov V. A. Shnyrova A. V. Zimmerberg J. (2011). Lipid polymorphisms and membrane shape. Cold Spring Harb. Perspect. Biol. 3:a004747. 10.1101/cshperspect.a004747 21646378 PMC3220359

[B15] Fujiwara H. Hosokawa M. Zhou X. Fujimoto S. Fukuda K. Toyoda K.. (2008). Curcumin inhibits glucose production in isolated mice hepatocytes. Diabet. Res. Clin. Pract. 80, 185–191. 10.1016/j.diabres.2007.12.004 18221818

[B16] Ge S. Yin T. Xu B. Gao S. Hu M. (2016). Curcumin affects phase II disposition of resveratrol through inhibiting efflux transporters MRP2 and BCRP. Pharm. Res. 33, 590–602. 10.1007/s11095-015-1812-1 26502886 PMC4744546

[B17] Guo F. Wu J. Wu W. Huang D. Yan Q. Yang Q.. (2018). PEGylated self-assembled enzyme-responsive nanoparticles for effective targeted therapy against lung tumors. J. Nanobiotechnol. 16:57. 10.1186/s12951-018-0384-8 30012166 PMC6048871

[B18] Hamano N. Böttger R. Lee S. E. Yang Y. Kulkarni J. A. Ip S.. (2019). Robust Microfluidic technology and new lipid composition for fabrication of curcumin-loaded liposomes: effect on the anticancer activity and safety of cisplatin. Mol. Pharm. 16, 3957–3967. 10.1021/acs.molpharmaceut.9b00583 31381352

[B19] Hanafy N. A. El-Kemary M. Leporatti S. (2018). Micelles structure development as a strategy to improve smart cancer therapy. Cancers 10:238. 10.3390/cancers10070238 30037052 PMC6071246

[B20] Hong S. S. Thapa R. K. Kim J. H. Kim S. Y. Kim J. O. Kim J. K.. (2018). Role of zein incorporation on hydrophobic drug-loading capacity and colloidal stability of phospholipid nanoparticles. Coll. Surf. B Biointerfaces 171, 514–521. 10.1016/j.colsurfb.2018.07.068 30096472

[B21] Huang S. He J. Cao L. Lin H. Zhang W. Zhong Q. (2020). Improved physicochemical properties of curcumin-loaded solid lipid nanoparticles stabilized by sodium caseinate-lactose maillard conjugate. J. Agric. Food Chem. 68, 7072–7081. 10.1021/acs.jafc.0c01171 32511914

[B22] Hussein Y. H. Youssry M. (2018). Polymeric micelles of biodegradable diblock copolymers: enhanced encapsulation of hydrophobic drugs. Materials 11:688. 10.3390/ma11050688 29702593 PMC5978065

[B23] Ireson C. R. Jones D. J. Orr S. Coughtrie M. W. Boocock D. J. Williams M. L.. (2002). Metabolism of the cancer chemopreventive agent curcumin in human and rat intestine. Cancer Epidemiol. Prev. Biomark. 11, 105–111. 11815407

[B24] Jenning V. Lippacher A. Gohla S. H. (2002). Medium scale production of solid lipid nanoparticles (SLN) by high pressure homogenization. J. Microencapsul. 19, 1–10. 10.1080/713817583 11811751

[B25] Kao H. H. Wu C. J. Won S. J. Shin J. W. Liu H. S. Su C. L. (2011). Kinase gene expression and subcellular protein expression pattern of protein kinase C isoforms in curcumin-treated human hepatocellular carcinoma Hep 3B cells. Plant Foods Hum. Nutr. 66, 136–142. 10.1007/s11130-011-0228-2 21556896

[B26] Karlsson J. Vaughan H. J. Green J. J. (2018). Biodegradable polymeric nanoparticles for therapeutic cancer treatments. Ann. Rev. Chem. Biomol. Eng. 9, 105–127. 10.1146/annurev-chembioeng-060817-084055 29579402 PMC6215694

[B27] Kesisoglou F. Panmai S. Wu Y. (2007). Nanosizing—oral formulation development and biopharmaceutical evaluation. Adv. Drug Deliv. Rev. 59, 631–644. 10.1016/j.addr.2007.05.003 17601629

[B28] Kumar A. Sirohi V. K. Anum F. Singh P. K. Gupta K. Gupta D.. (2017). Enhanced apoptosis, survivin down-regulation and assisted immunochemotherapy by curcumin loaded amphiphilic mixed micelles for subjugating endometrial cancer. Nanomedicine 13, 1953–1963. 10.1016/j.nano.2017.04.014 28457934

[B29] Lev-Ari S. Starr A. Vexler A. Karaush V. Loew V. Greif J.. (2006). Inhibition of pancreatic and lung adenocarcinoma cell survival by curcumin is associated with increased apoptosis, down-regulation of COX-2 and EGFR and inhibition of Erk1/2 activity. Anticancer Res. 26, 4423–4430. 17201164

[B30] Li X. Chen T. Xu L. Zhang Z. Li L. Chen H. (2014). Preparation of curcumin micelles and the *in vitro* and *in vivo* evaluation for cancer therapy. J. Biomed. Nanotechnol. 10, 1458–1468. 10.1166/jbn.2014.1840 25016646

[B31] Lopes-Rodrigues V. Sousa E. Vasconcelos M. H. (2016). Curcumin as a modulator of P-glycoprotein in cancer: challenges and perspectives. Pharmaceuticals 9:71. 10.3390/ph9040071 27834897 PMC5198046

[B32] Ma Z. Wang N. He H. Tang X. (2019). Pharmaceutical strategies of improving oral systemic bioavailability of curcumin for clinical application. J. Controll. Rel. 316, 359–380. 10.1016/j.jconrel.2019.10.053 31682912

[B33] Manjili H. K. Ghasemi P. Malvandi H. Mousavi M. S. Attari E. Danafar H. (2017). Pharmacokinetics and *in vivo* delivery of curcumin by copolymeric mPEG-PCL micelles. Eur. J. Pharm. Biopharm. 116, 17–30. 10.1016/j.ejpb.2016.10.003 27756682

[B34] Manjunath K. Reddy J. S. Venkateswarlu V. (2005). Solid lipid nanoparticles as drug delivery systems. Methods Find Exp. Clin. Pharmacol. 27, 127–144. 10.1358/mf.2005.27.2.876286 15834465

[B35] Mishra A. Kumar R. Tyagi A. Kohaar I. Hedau S. Bharti A. C.. (2015). Curcumin modulates cellular AP-1, NF-kB, and HPV16 E6 proteins in oral cancer. Ecancermedicalscience 9:525. 10.3332/ecancer.2015.525 25932049 PMC4407748

[B36] Moghimi S. M. Hunter A. C. Murray J. C. (2005). Nanomedicine: current status and future prospects. FASEB J. 19, 311–330. 10.1096/fj.04-2747rev 15746175

[B37] Mohanty C. Das M. Sahoo S. K. (2012). Emerging role of nanocarriers to increase the solubility and bioavailability of curcumin. Expert Opin. Drug Deliv. 9, 1347–1364. 10.1517/17425247.2012.724676 22971222

[B38] Nagaraju G. P. Benton L. Bethi S. R. Shoji M. El-Rayes B. F. (2019). Curcumin analogs: Their roles in pancreatic cancer growth and metastasis. Int J. Cancer 145, 10–19. 10.1002/ijc.31867 30226272

[B39] Oerlemans C. Bult W. Bos M. Storm G. Nijsen J. F. W. Hennink W. E. (2010). Polymeric micelles in anticancer therapy: targeting, imaging and triggered release. Pharm. Res. 27, 2569–2589. 10.1007/s11095-010-0233-4 20725771 PMC2982955

[B40] Papahadjopoulos D. Kimelberg H. K. (1974). Phospholipid vesicles (liposomes) as models for biological membranes: their properties and interactions with cholesterol and proteins. Progress Surf. Sci. 4, 141–232. 10.1016/S0079-6816(74)80006-7

[B41] Peng S. Li Z. Zou L. Liu W. Liu C. McClements D. J. (2018a). Enhancement of curcumin bioavailability by encapsulation in sophorolipid-coated nanoparticles: an *in vitro* and *in vivo* study. J. Agric. Food Chem. 66, 1488–1497. 10.1021/acs.jafc.7b05478 29378117

[B42] Peng S. Li Z. Zou L. Liu W. Liu C. McClements D. J. (2018b). Improving curcumin solubility and bioavailability by encapsulation in saponin-coated curcumin nanoparticles prepared using a simple pH-driven loading method. Food Func. 9, 1829–1839. 10.1039/C7FO01814B 29517797

[B43] Rutz J. Maxeiner S. Juengel E. Bernd A. Kippenberger S. Zöller N.. (2019). Growth and proliferation of renal cell carcinoma cells is blocked by low curcumin concentrations combined with visible light irradiation. Int. J. Mol. Sci. 20:1464. 10.3390/ijms20061464 30909499 PMC6471746

[B44] Sadegh Malvajerd S. Azadi A. Izadi Z. Kurd M. Dara T. Dibaei M.. (2018). Brain delivery of curcumin using solid lipid nanoparticles and nanostructured lipid carriers: Preparation, optimization, and pharmacokinetic evaluation. ACS Chem. Neurosci. 10, 728–739. 10.1021/acschemneuro.8b00510 30335941

[B45] Selvamuthukumar S. Velmurugan R. (2012). Nanostructured lipid carriers: a potential drug carrier for cancer chemotherapy. Lipids Health Dis. 11:159. 10.1186/1476-511X-11-159 23167765 PMC3561225

[B46] Seo J. Kim B. Dhanasekaran D. N. Tsang B. K. Song Y. S. (2016). Curcumin induces apoptosis by inhibiting sarco/endoplasmic reticulum Ca2^+^ ATPase activity in ovarian cancer cells. Cancer Lett. 371, 30–37. 10.1016/j.canlet.2015.11.021 26607901

[B47] Sharifi S. Fathi N. Memar M. Y. Hosseiniyan Khatibi S. M. Khalilov R. Negahdari R.. (2020). Anti-microbial activity of curcumin nanoformulations: new trends and future perspectives. Phytother. Res. 34, 1926–1946. 10.1002/ptr.6658 32166813

[B48] Song Z. Feng R. Sun M. Guo C. Gao Y. Li L.. (2011). Curcumin-loaded PLGA-PEG-PLGA triblock copolymeric micelles: preparation, pharmacokinetics and distribution *in vivo*. J. Colloid Interf. Sci. 354, 116–123. 10.1016/j.jcis.2010.10.024 21044788

[B49] Sreenivasan S. Ravichandran S. Vetrivel U. Krishnakumar S. (2012). *In vitro* and *in silico* studies on inhibitory effects of curcumin on multi drug resistance associated protein (MRP1) in retinoblastoma cells. Bioinformation 8:13. 10.6026/97320630008013 22359429 PMC3282270

[B50] Stridh M. H. Correa F. Nodin C. Weber S. G. Blomstrand F. Nilsson M.. (2010). Enhanced glutathione efflux from astrocytes in culture by low extracellular Ca2^+^ and curcumin. Neurochem. Res. 35, 1231–1238. 10.1007/s11064-010-0179-2 20437093 PMC3097133

[B51] Sun D. Zhou J. K. Zhao L. Zheng Z. Y. Li J. Pu W.. (2017). Novel curcumin liposome modified with hyaluronan targeting CD44 plays an anti-leukemic role in acute myeloid leukemia *in vitro* and *in vivo*. ACS Appl. Mater. Interf. 9, 16857–16868. 10.1021/acsami.7b02863 28489348

[B52] Sun M. Su X. Ding B. He X. Liu X. Yu A.. (2012). Advances in nanotechnology-based delivery systems for curcumin. Nanomedicine 7, 1085–1100. 10.2217/nnm.12.80 22846093

[B53] Tapeinos C. Battaglini M. Ciofani G. (2017). Advances in the design of solid lipid nanoparticles and nanostructured lipid carriers for targeting brain diseases. J. Control. Release 264, 306–332. 10.1016/j.jconrel.2017.08.033 28844756 PMC6701993

[B54] Thangapazham R. L. Puri A. Tele S. Blumenthal R. Maheshwari R. K. (2008). Evaluation of a nanotechnology based carrier for delivery of curcumin in prostate cancer cells. Int. J. Oncol. 32, 1119–1123. 10.3892/ijo.32.5.1119 18425340 PMC2547884

[B55] Tian C. Asghar S. Wu Y. Chen Z. Jin X. Yin L.. (2017a). Improving intestinal absorption and oral bioavailability of curcumin via taurocholic acid-modified nanostructured lipid carriers. Int. J. Nanomed. 12:7897. 10.2147/IJN.S145988 29138557 PMC5667785

[B56] Tian C. Asghar S. Wu Y. Kambere Amerigos D. Chen Z. Zhang M.. (2017b). N-acetyl-L-cysteine functionalized nanostructured lipid carrier for improving oral bioavailability of curcumin: preparation, *in vitro* and *in vivo* evaluations. Drug Deliv. 24, 1605–1616. 10.1080/10717544.2017.1391890 29063815 PMC8241171

[B57] Tian M. P. Song R. X. Wang T. Sun M. J. Liu Y. Chen X. G. (2018). Inducing sustained release and improving oral bioavailability of curcumin via chitosan derivatives-coated liposomes. Int. J. Biol. Macromol. 120, 702–710. 10.1016/j.ijbiomac.2018.08.146 30170061

[B58] Tønnesen H. H. Másson M. Loftsson T. (2002). Studies of curcumin and curcuminoids. XXVII. Cyclodextrin complexation: solubility, chemical and photochemical stability. Int. J. Pharm. 244, 127–135. 10.1016/S0378-5173(02)00323-X 12204572

[B59] Torchilin V. P. (2007). Micellar nanocarriers: pharmaceutical perspectives. Pharm. Res. 24:1. 10.1007/s11095-006-9132-0 17109211

[B60] Weber S. Zimmer A. Pardeike J. (2014). Solid lipid nanoparticles (SLN) and nanostructured lipid carriers (NLC) for pulmonary application: a review of the state of the art. Eur. J. Pharm. Biopharm. 86, 7–22. 10.1016/j.ejpb.2013.08.013 24007657

[B61] Wei X. Senanayake T. H. Bohling A. Vinogradov S. V. (2014). Targeted nanogel conjugate for improved stability and cellular permeability of curcumin: synthesis, pharmacokinetics, and tumor growth inhibition. Mol. Pharm. 11, 3112–3122. 10.1021/mp500290f 25072100 PMC4151794

[B62] Willenbacher E. Khan S. Z. Mujica S. C. A. Trapani D. Hussain S. Wolf D.. (2019). Curcumin: new insights into an ancient ingredient against cancer. Int. J. Mol. Sci. 20:1808. 10.3390/ijms20081808 31013694 PMC6514995

[B63] Wu W. Wu J. Fu Q. Jin C. Guo F. Yan Q.. (2019). Elaboration and characterization of curcumin-loaded Tri-CL-mPEG three-arm copolymeric nanoparticles by a microchannel technology. Int. J. Nanomed. 14, 4683–4695. 10.2147/IJN.S198217 31308653 PMC6615023

[B64] Xu H. Gong Z. Zhou S. Yang S. Wang D. Chen X.. (2018). Liposomal curcumin targeting endometrial cancer through the nf-κB pathway. Cell. Physiol. Biochem. 48, 569–582. 10.1159/000491886 30021217

[B65] Yallapu M. M. Jaggi M. Chauhan S. C. (2012). Curcumin nanoformulations: a future nanomedicine for cancer. Drug Disc. Today 17, 71–80. 10.1016/j.drudis.2011.09.009 21959306 PMC3259195

[B66] Yedjou C. G. Mbemi A. T. Noubissi F. Tchounwou S. S. Tsabang N. Payton M.. (2019). Prostate cancer disparity, chemoprevention, and treatment by specific medicinal plants. Nutrients 11:336. 10.3390/nu11020336 30720759 PMC6412894

[B67] Zhang H. Y. Sun C. Adu-Frimpong M. Yu J. Xu X. (2019). Glutathione-sensitive PEGylated curcumin prodrug nanomicelles: preparation, characterization, cellular uptake and bioavailability evaluation. Int. J. Pharm. 555, 270–279. 10.1016/j.ijpharm.2018.11.049 30471374

[B68] Zhao J. Liu J. Xu S. Zhou J. Han S. Deng L.. (2013). Graft copolymer nanoparticles with pH and reduction dual-induced disassemblable property for enhanced intracellular curcumin release. ACS Appl. Mater. Interf. 5, 13216–13226. 10.1021/am404213w 24313273

[B69] Zhu G. H. Dai H. P. Shen Q. Ji O. Zhang Q. Zhai Y. L. (2016). Curcumin induces apoptosis and suppresses invasion through MAPK and MMP signaling in human monocytic leukemia SHI-1 cells. Pharm. Biol. 54, 1303–1311. 10.3109/13880209.2015.1060508 26134921

